# Membrane Functionalization with Hyperbranched Polymers

**DOI:** 10.3390/ma9080706

**Published:** 2016-08-20

**Authors:** Agnes Schulze, Marco Went, Andrea Prager

**Affiliations:** Leibniz Institute of Surface Functionalization, Permoserstr. 15, Leipzig D-04318, Germany; marco.went@iom-leipzig.de (M.W.); andrea.prager@iom-leipzig.de (A.P.)

**Keywords:** polymer membranes, hyperbranched polymers, surface functionalization, zeta potential, protein adsorption

## Abstract

Polymer membranes have been modified with hyperbranched polymers with the aim to generate a high density of hydrophilic functional groups at the membrane surface. For this purpose hyperbranched polymers containing amino, alcohol, and carboxylic acid end groups were used for membrane modification, respectively. Thus, surface potential and charges were changed significantly to result in attractive or repulsive interactions towards three different proteins (albumin, lysozyme, myoglobin) that were used to indicate membrane fouling properties. Our studies demonstrated that hydrophilization alone is not effective for avoiding membrane fouling when charged proteins are present. In contrast, electrostatic repulsion seems to be a general key factor.

## 1. Introduction

Modern separation technologies such as waste water treatment, sterilization filtration, hemodialysis, the production of fine chemicals, processes of the dairy industry, etc., are predominantly based on using porous polymer membranes [1]. The required process conditions make great demands on the chemical and physical stability of the membrane material itself. Therefore, polymer membranes are fabricated from robust synthetic materials such as polyethersulfone (PES), polysulfone (PSf), or polyvinylidene fluoride (PVDF) which offer high stability within a broad range of process conditions [2]. However, membranes made from these polymers are prone to fouling which is caused by hydrophobic interactions of the membrane surface with biomolecules or colloids in the mixture to be filtered and result in irreversible adsorption, aggregation, ripening, and finally in a reduced filtration performance [3,4,5,6].

To reduce the problem of initial fouling, different approaches for surface hydrophilization have been investigated such as copolymerization or grafting with hydrophilic monomers [7,8,9,10,11,12,13], small organic molecules [14,15], polymers [16], enzymes [17,18]; blending using hydrophilic polymers [19,20,21,22,23,24]; incorporation of inorganic materials [25,26]; and, finally, chemical modification of the membrane polymer [27].

However, not many approaches have been proposed using hyperbranched polymers or dendritic structures to generate hydrophilic groups on membranes surfaces, although these structures should offer a high density of functional groups at the membrane surface. So far, hyperbranched polymers have been introduced into the membrane material to impact gas permeation properties [28,29,30,31,32]. Diverse methods have been developed to create hyperbranched polymer structures on top of polymer surfaces including neutral, alkaline, or acidic end groups [33,34,35,36,37,38,39,40]. Since some of these structures can be generated in a step-wise manner by growing generations successively, the density of functional groups can be controlled by the number of generations that is developed.

**Figure 1:**
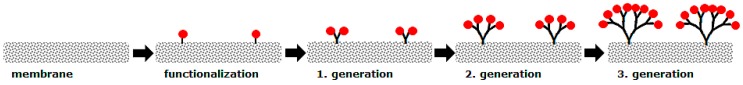
Schematic of membrane surface modification by growing hyperbranched polymeric structures.

In the present work we discuss the functionalization of a hydrophobic PVDF membrane surface with hyperbranched polymers to impact the fouling properties. The use of hyperbranched polymers leads to a high density of functional groups on the membrane surface (see Figure 1). We equipped the membrane with hydrophilic neutral (alcohol groups), alkaline (amino groups), and acidic (carboxylic groups) hyperbranched polymers, and investigated the resulting membrane performance by determination of protein adsorption. Further characterization was done by contact angle measurements, determination of the surface potential, XPS, pure water permeation flux, porosimetry, and SEM.

## 2. Results and Discussion

### 2.1. Membrane Surface Functionalization

**Figure 2:**
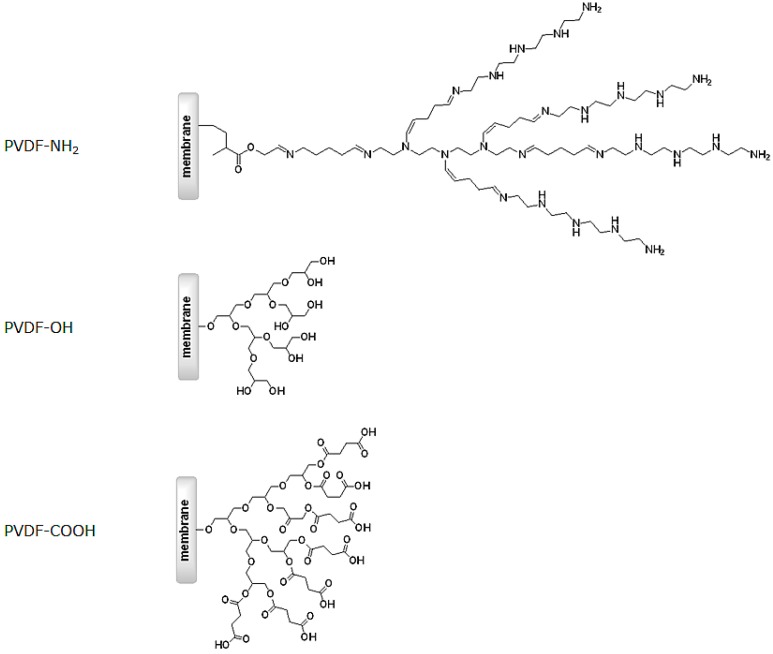
Three different hyperbranched polymer structures were synthesized on top of PVDF membranes to result in a high density of alkaline, neutral and acidic functional groups on the membrane surface.

PVDF microfiltration membranes have been modified with three different hydrophilic hyperbranched polymer systems to result in PVDF-NH_2_, PVDF-OH, and PVDF-COOH (Figure 2). First, amino groups containing hyperbranched polymers were generated in a step-wise synthesis with control over the generation growth (for experimental details see Section 3.2). The inert PVDF membrane surface was initially modified by electron beam–based grafting with aminoethyl methacrylate followed by a repeated reaction with glutaraldehyde and tetraethylenepentamine, subsequently. Thus, up to four generations of amino group–based hyperbranched structures were generated on the membrane surface (Figure 2 represents the second generation of PVDF-NH_2_). For preparation of the PVDF-OH membrane, surface activation was performed by plasma treatment followed by reaction with glycidol to form a neutral hyperbranched structure. The growth could only be controlled by the variation of time since the growth mechanism follows a polymerization reaction. This type of membrane was then also used to transform it into the PVDF-COOH membrane by further reaction with pyridine and succinic acid.

**Figure 3:**
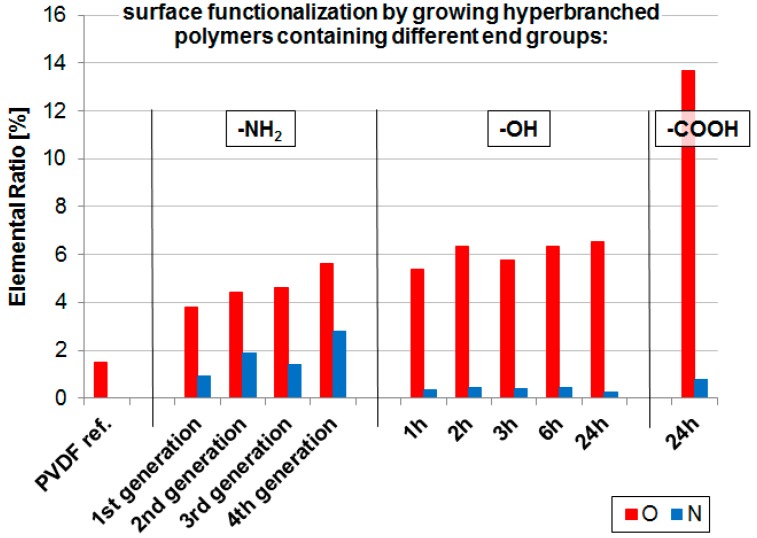
Results of XPS analysis of the pristine PVDF membrane (PVDF ref.) and after growth of hyperbranched polymers on top of the membrane surface (PVDF-NH_2_, PVDF-OH, PVDF-COOH). PVDF-NH_2_ membranes were prepared in controlled steps to gain defined generations, while PVDF-OH membranes were prepared via a polymerization reaction which was stopped after different times (1–24 h). PVDF-COOH was prepared by further reaction of the PVDF-OH membrane (24 h).

The growth of the hyperbranched polymers was investigated by XPS analysis (Table 1 and Figure 3). The step-wise growth of hyperbranched amino-containing structures by growing up to four generations was demonstrated by the N content of the respective PVDF-NH_2_ membranes. While no N was detected in case of the reference PVDF membrane, the N ratio was successively increased to reach 0.9%–2.8% for generations 1–4 (Table 1). Since the O content was also increasing, we assumed that the conversion with glutaraldehyde was not 100% completed, probably due to increasing the steric hindrance of the hyperbranched structure. While N and O content increased with the number of generations, the content of F decreased (reference membrane: 47%, generations 1–4: 43%–35%). This can be explained with the formation of a layer of the hyperbranched polymer on top on the membrane surface. In XPS analysis the penetration depth is adjusted to reach only the first nanometers of the surface. Therefore, the F content which can be assigned to the PVDF membrane material is covered by the growing hyberbranched polymer and appears to decrease.

In contrast, the polymerization of glycerol did not result in a time-dependent formation of PVDF-OH membranes, and the resulting O content is comparable regarding the different reaction times of 1–24 h (Table 1 and Figure 3). The reaction seems to be very fast to be completed within 1 h, so no defined generations could be generated. Similar to the formation of PVDF-NH_2_ membranes, we found a decrease in F resulting from the successful growth of a hyperbranched polymer on the PVDF-OH membrane surface. Since the last membrane type—PVDF-COOH—was prepared by further reaction of the PVDF-OH membrane, we decided to use that membrane which was prepared by the glycidol reaction within 24 h since no significant differences were observed within the different time-dependent reactions. Therefore, only one hyperbranched PVDF-COOH membrane was prepared. The formation of COOH groups was confirmed by XPS analysis by the general increase in O content (13.7% for PVDF-COOH in contrast to 6.6% for PVDF-OH) and by the appearance of a new C-O bond at 297.2 eV with an intensity of 9.1% in the C1s deconvolution analysis (Table 1). Furthermore, the F content was further decreased with this additional reaction step from 42% (PVDF-OH 24 h) to 31% (PVDF-COOH).

**Figure 4:**
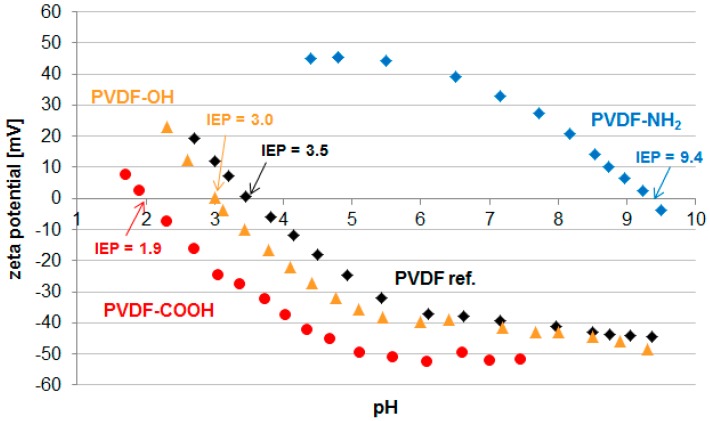
Zeta potential vs. pH of the pristine and modified PVDF membranes including the corresponding isoelectric points (IEP).

The surface functionalization was then characterized by the determination of the zeta potential (Figure 4). Characterization of the functionalized membranes was conducted using the PVDF-NH_2_ after the fourth generation of growth, and PVDF-OH and PVDF-COOH after 24 h of polymerization time. Although the pristine PVDF membrane possesses no charged groups, an isoelectric point (IEP) of 3.5 was detected. Titration within a neutral and alkaline medium revealed a negative charge on the surface of the pristine PVDF membrane (−39 mV at pH 7). This trend has already been reported for uncharged polymers and can be explained with the adsorption of hydroxide ions originating from the self-ionization of water [41,42,43]. Therefore, the PVDF membrane surface appears to be negatively charged.

Regarding the PVDF-NH_2_ membrane, the generation of alkaline functional groups was confirmed. A positive charge (+33 mV at pH 7) was detected in acidic and neutral medium and an IEP of 9.4 was determined as expected. In the case of the neutral PVDF-OH membrane, the zeta potential vs. pH data is comparable to the PVDF ref. membrane. This is in accordance with the above discussed behavior of neutral polymer surfaces since no charged groups have been generated on the membrane surface. Finally, acidic groups were synthesized to gain the PVDF-COOH membrane, and the surface therefore possesses a negative charge (−52 mV at pH 7) accompanied by an IEP of 1.9.

In summary, the successful growth of hyperbranched polymers carrying alkaline, neutral, and acidic functional groups has been confirmed by XPS and zeta potential analysis.

### 2.2. Membrane Properties

**Figure 5:**
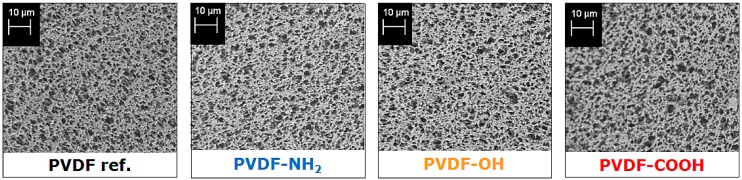
SEM images of the pristine PVDF membrane (PVDF ref.) and after growth of hyperbranched polymers on top of the membrane surface (PVDF-NH_2_, PVDF-OH, PVDF-COOH).

Regarding the pore morphology by SEM, no significant changes (e.g., pore blocking or destruction) are visible due to the growth of hyperbranched polymers on the PVDF membranes (Figure 5). However, SEM images can just give a first impression since only the surface morphology is presented. Even marginal changes could impact the total membrane porosity and, thus, performance characteristics such as pure water flux.

Determination of the pure water flux of the modified membranes revealed a slight performance decrease (Table 2). While the pristine PVDF membrane had a water permeation flux of 27,605 L/(h·m²·bar) the growth of hyperbranched structures on top of the membrane surface led to decreased values. This can be explained with the formation of an additional polymer layer on the membrane surface as well as in the pores (see Section 3.2) which reduces the pore diameter, and therefore, the water permeation flux is reduced compared to the unmodified membrane. Concerning the water permeation flux, the largest impact was determined regarding the PVDF-COOH membrane with a flux of 74% (20,539 L/(h·m²·bar)) compared to the pristine PVDF membrane.

The determination of the static water contact angle is a common method for the determination of the relative surface wettability of a membrane. The pristine PVDF membrane is hydrophobic, and a water contact angle of 119.8° was determined. The growth of hyperbranched hydrophilic polymers resulted in an improved water wettability. The largest impact was achieved for the PVDF-NH_2_ membrane with an improvement of 31% (82.8°). Surprisingly, the change of the water contact angle of the PVDF-OH membrane was not very large (−2.4° compared to the pristine PVDF membrane), although a lot of hydrophilic hydroxyl groups have been generated as confirmed by XPS analysis (Table 1 and Figure 3). However, an improved water wettability was observed in our lab experiments since the water uptake in water permeation flux experiments could be clearly observed as also confirmed by simple visualization (the PVDF membrane becomes opaque when wetted with water). We attribute this fact again to the layer of the hyperbranched polymer covering the membrane's surface, and therefore, impacting the pore size, roughness, and/or capillary forces. All mentioned parameters can influence the resulting water contact angle, making a direct comparison with the pristine membrane difficult.

**Figure 6:**
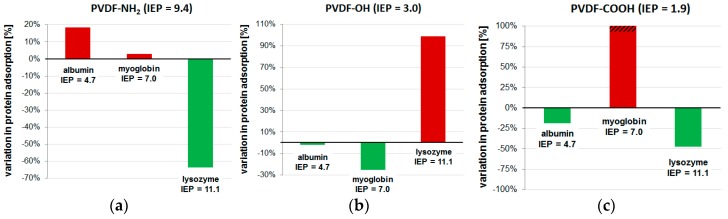
Variations in protein adsorption at the surface of the three modified PVDF membranes: (**a**) PVDF-NH_2_; (**b**) PVDF-OH; (**c**) PVDF-COOH relative to the pristine PVDF membrane. The proteins used in the test were albumin, myoglobin, and lysozyme.

The main focus of our study was the investigation of differently modified membrane surfaces regarding the resulting protein adsorption. We used three different proteins with different overall charges: albumin (IEP = 4.7), myoglobin (IEP = 7.0), and lysozyme (IEP = 11.1). The results of the protein adsorption tests are presented in Table 2 and Figure 6.

Although all three membranes have been modified with hydrophilic hyperbranched polymer structures, we found no general decrease in protein adsorption. This can be explained by considering the electrostatic interactions of the proteins with differently charged membranes. The protein adsorption test is performed at pH in phosphate buffered saline (without any multivalent cations, e.g., Ca^2+^). The PVDF-NH_2_ membrane has an IEP of 9.4 and is positively charged at pH (Figure 4). Compared to the pristine PVDF membrane, the adsorption of albumin is increased (+18%) while lysozyme adsorption was significantly decreased (−64%). Since albumin is a negatively charged protein, electrostatic attraction can occur towards the positively charged PVDF-NH_2_ membrane. In contrast, electrostatic repulsion of the positively charged lysozyme explains the decreased adsorption on this membrane. Opposite results have been found for the PVDF-OH membrane which is negatively charged at pH 7 (Figure 4). Here, albumin and myoglobin adsorption decreased (−1% and −25%) while lysozyme adsorption increased significantly (+99%). Finally, the negatively charged PVDF-COOH membrane showed increased adsorption of myoglobin while albumin adsorption was successfully decreased (−19%). Surprisingly, the adsorption of lysozyme was also decreased in this case. This cannot be explained by only regarding electrostatic repulsion/attraction or hydrophilicity. Furthermore, myoglobin adsorption was very high (+644%). Possibly, these unexpected effects can be explained by conformation changes when proteins are adsorbed to an acidic surface. Others have already reported that proteins can unfold and lose their active structure while fouling a membrane [44]. Thus, the protein surface charge may be changed and, therefore, the adsorption results were not comparable with those obtained in the experiments with the other membrane surfaces. However, our experiments revealed that electrostatic repulsion is effective for all tested proteins in decreasing their adsorption, and in the case of membrane modification with amino or alcohol groups, electrostatic attraction leads to increased protein adsorption.

## 3. Materials and Methods

### 3.1. Chemicals and Materials

Poly(vinylidene fluoride) membranes (hydrophobic, pore size 0.45 μm, thickness 125 μm) were purchased from Carl Roth GmbH & Co. (Karlsruhe, Germany). Tetraethylenepentamine, ethanol, bovine serum albumin (fraction V, pH 5) (isoelectric point (IEP) = 4.7, molecular mass = 67,200 Da, acidic protein), myoglobin from equine skeletal muscle (95%–100%, essentially salt-free, lyophilized powder, IEP = 7.0, 17,800 Da, neutral protein), lysozyme from chicken egg white (lyophilized powder, protein 90%, 40,000 units/mg of protein, IEP = 11.1, 14,600 Da, basic protein), glycidol, pyridine (water-free), succinic anhydride were purchased from Sigma-Aldrich (Steinheim, Germany). Other purchased chemicals: 2-aminoethyl methacrylate hydrochloride (Acros Organics, Waltham, MA, USA), glutaraldehyde (Merck, Kenilworth, NJ, USA), hydrochloric acid solution (0.1 M, VWR), sodium hydroxide solution (0.1 M, VWR), sodium carbonate (anhydrate, VWR), sodium bicarbonate (Waltham, MA, USA). Bicinchoninic acid (BCA) protein assay reagent A + B was provided by Pierce (Rockford, IL, USA). Phosphate-buffered saline (PBS; 50 mM) was used at pH 7. If not otherwise stated Millipore^®^ grade water was used. All chemicals were of analytical grade and used without further purification.

### 3.2. Membrane Surface Functionalization with Hyperbranched Polymers

To generate alkaline hyperbranched polymers on their surface membranes were dipped into an aqueous solution of aminoethyl methacrylate hydrochloride (0.5 wt. %), followed by electron beam irradiation and rinsing with water three times for 30 min in wet state. Irradiation was performed in a N_2_ atmosphere with O_2_ quantities <10 ppm using a custom-made electron accelerator [45], and an irradiation dose of 150 kGy. The voltage and the current were set to 160 kV and 10 mA, respectively. The absorbed dose was adjusted by the speed of the sample transporter. Since the electron irradiation is able to interpenetrate the entire cross-section of the membrane, modification will take place not only at the upper membrane surface but also within the pores [14]. Then, membranes were dipped into an aqueous solution of glutaraldehyde (2 wt. %) at pH 9.2 (NaHCO_3_/Na_2_CO_3_ buffer system) for 2 h. Glutaraldehyde solution was removed and the membrane was roughly rinsed before immersing into an aqueous solution of tetraethylenepentamine (2 wt. %) at pH 9.2 (NaHCO_3_/Na_2_CO_3_ buffer system) for another 2 h. The reactions with glutaraldehyde and tetraethylenepentamine were repeated as described before to create up to four generations of dendrimer structures [37].

Neutral hyperbranched polymers were created on the membrane by first activating the surface using oxygen plasma using a 300 W Junior Plasma System, Europlasma NV, Oudenaarde, Belgium for 5 min. After plasma treatment, the samples were in contact with ambient air, within 30 min they were directly placed into a solution of glycidol for 1–24 h at room temperature [38].

To generate acidic hyperbranched polymers the former modified membranes (Ø 47 mm) with glycidol were further treated using pyridine (10 mL) under exclusion of water. Then, 5 g of succinic anhydride was added, and the solution was stirred at 65 °C for 24 h under argon in the dark [35].

After modification, all samples were thoroughly rinsed three times with water, once with ethanol, and finally air dried in a dust free environment.

### 3.3. Membrane Characterization

The morphology of the porous materials was studied by scanning electron microscopy (SEM, Ultra 55, Carl Zeiss SMT, Jena, Germany). In order to prevent charging the sample was sputtered with a thin gold layer.

Chemical composition was analyzed with X-ray photoelectron spectroscopy (AXIS Ultra, Kratos Analytical, Manchester, UK). The kinetic energy of the electrons was analyzed with a pass energy of 160 eV for the survey spectra and 40 eV for the energy resolved spectra, respectively.

The water permeation flux was examined with a stainless steel pressure filter holder (16249, Sartorius, Germany) for dead-end filtration. The membrane (active area: 17.35 cm^2^) was compacted with 0.5 bar and ultrapure water (100 mL). The time was taken and the water permeation flux was calculated with following equation:

(1) $$J=\frac{V}{t\cdot A\cdot p}$$

where *J* is the permeation flux (mL·min^−1^·cm^−2^·bar); *V* is the volume (mL); *t* is time (min); *A* is the surface area of the membrane (cm^2^) and *p* is the pressure (bar).

Protein adsorption on the membranes were investigated using the bicinchoninic acid based assay (Pierce) [46] according to a before described method [14]. Therefore, the samples were washed three times with 1 mL of PBS buffer solution (pH 7). Then, the BCA reagent was added to the membrane samples and the plate was incubated for 25 min at 37 °C. The plate was then shaken for 5 min at room temperature, the solution was transferred to a new microtiter plate and light adsorption at 562 nm was measured using a microtiter plate reader (Infinite M200, Tecan, Crailsheim, Germany). For calibration, seven protein concentrations of 1000, 500, 250, 125, 62.50, 31.25 and 0.00 µg/mL were used.

Static water contact angle measurements were carried out on a DSA II (Krüss, Hamburg, Germany). Before measuring, a piece of the membrane (30 × 9 mm) was pressed to a dense film in order to eliminate the effect of capillary forces [47]. A 5 ml water drop was placed onto the pressed membrane with a microsyringe. At least 10 contact angles per five different locations were averaged.

Membrane zeta potentials were determined using streaming potential measurements carried out with the adjustable gap cell in the SurPASS system (Anton Paar, Graz, Austria), where the zeta potential *ζ* can be calculated based on the Smoluchowski equation given in Equation (2).

(2) $$\zeta =\frac{dU}{dp}\cdot \frac{\eta }{\varepsilon \cdot \varepsilon_{0}}\cdot \kappa$$

where *U* is the streaming potential; *p* the pressure; *η* the viscosity of the electrolyte solution; *ε* the dielectric constant of the electrolyte solution; *ε*_0_ the vacuum permittivity and *κ* the electrolyte conductivity. The corresponding charge density *σ* was calculated according to the following equation [48]:

(3) $$\sigma =\sqrt{8\cdot c_{0}\cdot \varepsilon \cdot \varepsilon_{0}\cdot R\cdot T}\cdot \sin h\left(\frac{z\cdot \varepsilon \cdot \psi_{0}}{2k_{B}\cdot T}\right)$$

where *c*_0_ is the concentration of the electrolyte; *R* is the ideal gas constant; *T* is temperature; *z* is the ion valency; $\psi_{0}$ is the surface potential and *k_B_* is the Boltzmann constant.

## 4. Conclusions

PVDF membranes have been modified by growing three different hyperbranched polymers containing alkaline, neutral, and acidic functional groups (-NH_2_, -OH, COOH) to obtain a membrane surface with a high density of hydrophilic groups. The successful surface modification was confirmed by XPS and zeta potential analysis. Membrane surface potential and charges were changed significantly to result in attractive or repulsive interactions towards three different proteins (albumin, lysozyme, myoglobin) which were used to indicate membrane fouling properties. Our studies demonstrated that hydrophilization alone is not effective for avoiding membrane fouling when charged proteins are present. In contrast, electrostatic repulsion seems to be a general key factor. However, membranes possessing an acidic surface showed unexpected fouling which was not explicable considering hydrophilicity or electrostatic attraction. In this case, we argue with pH-dependent protein denaturation or conformation changes that influence the protein surface charge. Therefore, future studies should consider fouling test systems with static/inert reagents such as nanoparticles possessing different surface charges to enable a direct comparison of electrostatic interactions between the membrane surface and fouling reagent.

## Figures and Tables

**Table 1:** XPS data of the pristine and modified PVDF membranes. PVDF-NH_2_ membranes were prepared in controlled steps to gain defined generations (1–4), while PVDF-OH membranes were prepared via a polymerization reaction which was stopped after different times (1–24 h). PVDF-COOH was prepared by further reaction of the PVDF-OH membrane (24 h).

Sample	Elemental Ratio [mol %]				C1s Deconvolution [eV; mol %]				
	C 1s	F 1s	O 1s	N 1s	284.7 C-C	286.4 -CH_2_-	287.2 C-O	288.4 C-O_x_	290.9 -CF_2_-
**PVDF ref.**	51.5	47.0	1.5	-	5.7	23.6	-	1.0	21.2
**PVDF-NH_2_ 1st**	52.5	42.8	3.8	0.9	6.1	24.3	-	3.7	19.3
**PVDF-NH_2_ 2nd**	54.2	39.5	4.4	1.9	7.6	26.5	-	5.0	17.2
**PVDF-NH_2_ 3rd**	53.4	40.6	4.6	1.4	4.1	25.4	-	3.3	19.8
**PVDF-NH_2_ 4th**	56.3	35.3	5.6	2.8	4.9	26.6	-	4.1	18.6
**PVDF-OH 1 h**	49.4	44.9	5.4	0.4	0.6	23.5	-	4.5	21.0
**PVDF-OH 2 h**	49.7	43.6	6.4	0.5	1.6	24.1	-	5.0	19.1
**PVDF-OH 3 h**	50.5	43.4	5.8	0.4	1.6	23.8	-	5.7	19.5
**PVDF-OH 6 h**	50.7	42.6	6.4	0.5	2.0	24.8	-	4.7	19.3
**PVDF-OH 24 h**	51.7	41.6	6.6	0.3	2.9	25.7	-	4.6	18.6
**PVDF-COOH**	55.0	30.5	13.7	0.8	7.9	19.5	9.1	5.1	13.4

**Table 2:** Performance data of the pristine and modified PVDF membranes regarding water permeation flux, water contact angle, and protein adsorption (albumin, myoglobin, and lysozyme).

Sample	Water Permeation Flux	Water Contact Angle	Protein Adsorption [µg/cm²]		
	[L/(h·m²·bar)]	[°]	Albumin IEP = 4.7	Myoglobin IEP = 7.0	Lysozyme IEP = 11.1
**PVDF ref.**	27,605	119.8 ± 3.6	20.8 ± 1.0	21.4 ± 1.0	25.0 ± 2.1
**PVDF-NH_2_**	24,431	82.8 ± 3.6	24.6 ± 6.7	22.0 ± 2.3	9.1 ± 2.5
**PVDF-OH**	22,814	116.4 ± 0.0	20.5 ± 0.8	16.1 ± 1.2	49.7 ± 1.9
**PVDF-COOH**	20,539	109.9 ± 0.0	16.9 ± 1.5	159.3 ± 16.2	13.2 ± 2.9
